# Mangiferin Attenuates LPS/D-GalN-Induced Acute Liver Injury by Promoting HO-1 in Kupffer Cells

**DOI:** 10.3389/fimmu.2020.00285

**Published:** 2020-02-25

**Authors:** Sen Yang, Ge Kuang, Liangke Zhang, Shengwang Wu, Zizuo Zhao, Bin Wang, Xinru Yin, Xia Gong, Jingyuan Wan

**Affiliations:** ^1^Chongqing Key Laboratory of Biochemistry and Molecular Pharmacology, Chongqing Medical University, Chongqing, China; ^2^Department of Anatomy, Chongqing Medical University, Chongqing, China; ^3^Department of Anesthesiology, The First Affiliated Hospital of Chongqing Medical University, Chongqing, China; ^4^Department of Gastroenterology, Institute of Surgery Research, Daping Hospital, Third Military Medical University, Chongqing, China

**Keywords:** mangiferin, heme oxygenase-1 (HO-1), acute liver injury, lipopolysaccharide/D-galactosamine (LPS/D-GalN), TNF-α

## Abstract

Acute liver injury and its terminal phase, hepatic failure, trigger a series of complications, including hepatic encephalopathy, systematic inflammatory response syndrome, and multiorgan failure, with relatively high morbidity and mortality. Liver transplantation is the ultimate intervention, but the shortage of donor organs has limited clinical success. Mangiferin (MF), a xanthone glucoside, has been reported to have excellent anti-inflammatory efficacy. Here, a lipopolysaccharide (LPS)/D-galactosamine (D-GalN)-induced acute liver injury mouse model was established to investigate the protective role of MF and the underlying mechanisms of action. Pretreatment with MF improved survival, decreased serum aminotransferase activities, and inhibited hepatic TNF-α production in LPS/D-GalN-challenged mice. Through Kupffer cell (KC) deletion by GdCl_3_ and KC adoptive transfer, KCs were confirmed to be involved in these beneficial effects of MF. MF reduced LPS-mediated TNF-α production via the suppression of the TLR4/NF-κB signaling pathway *in vitro*. MF promoted HO-1 expression, but the knockdown of HO-1 prevented TNF-α inhibition, suggesting that the damage-resistance effects of HO-1 occurred via the suppression of TNF-α synthesis. When HO-1-silenced KCs were transferred to the liver with KC deletion, the protective effect of MF against LPS/D-GalN-induced acute liver injury was reduced, illustrating the role of KC-derived HO-1 in the anti-injury effects of MF. Collectively, MF attenuated acute liver injury induced by LPS/D-GalN via the inhibition of TNF-α production by promoting KCs to upregulate HO-1 expression.

## Introduction

The liver is the most important metabolic organ and plays a pivotal role in the host's defensive response owing to its ability to scavenge pathogenic microorganisms and toxins (1). However, the liver is also the main victim of such attacks, resulting in the activation of host immune cells, which incites inflammation (2). The dysregulated inflammatory response not only impairs the defensive function of liver but also induces massive necrosis of hepatocytes, triggering acute liver injury and, ultimately, provoking fulminant hepatic failure (FHF) (3, 4). As the incidence of FHF is associated with high mortality and there is no recognized treatment, the terminal stage of FHF should be prevented. Thus, there is an urgent need for new therapeutic agents for the clinical treatment of acute liver injury (5).

Lipopolysaccharide, a component of Gram-negative bacteria, can induce acute liver injury with high lethality in D-galactosamine (D-GalN)-sensitized mice (6). This well-established experimental murine model, developed by Galanos et al. (7), is distinguished by a dramatic activation of Kupffer cells (KCs), the resident macrophages of the liver tissue (8), which can produce many pro-inflammatory mediators in the liver (9). Among these mediators, TNF-α, the terminal effector in the pathogenesis of acute liver injury, initiates extensive hepatocyte apoptosis and massive hepatocyte necrosis, prompting the explosion of inflammation. Considering that the model exactly resembles the pathogenesis of human clinical FHF, it is employed widely as an experimental animal model to investigate the underlying mechanism of clinical FHF and develop effective therapeutic strategies for endotoxin challenge (10).

Heme oxygenase-1 (HO-1), a cellular stress protein belonging to the heme oxygenase enzyme system, is part of the rate-limiting step in the degradation of heme to carbon monoxide (CO), iron, and biliverdin-IXa (BV); all of these are involved in various pathophysiological processes (11). It has manifold biological activities, such as anti-oxidative stress, anti-inflammatory, and anti-apoptosis activity (12, 13). HO-1, a downstream molecule in multiple signaling pathways, is induced and responds to pathological challenges from various diverse stimuli (14). Recent studies have demonstrated that expression of HO-1 is upregulated in acute liver injury and that the increase in HO-1 activity alleviated the toxic action of LPS in the liver (15). In particular, the stimulation of macrophage-derived HO-1 appears to significantly attenuate liver injury, which has caused researchers to recognize its critical role (16).

Mangiferin, a xanthone glucoside, exhibits a broad spectrum of pharmacological properties, including antioxidant, antiviral, antitumor, anti-inflammatory, and gene-regulatory effects (17, 18). A growing number of studies have reported that MF exerts potent anti-oxidative and anti-inflammatory effects against the acute inflammation of multiple organs, such as the lung and kidney and the cardiovascular system (19–21). The attenuation of liver injury by MF via multiple pathways has been previously reported, illustrating its prominent role in hepatic inflammation (22, 23). With respect to the signaling pathways concerning anti-inflammation, Nrf2/HO-1 exerts its defense against injury by abating oxidative stress, mitigating the inflammatory response, and preventing cell death (24). Pan et al. (25) reported that MF alleviated LPS/D-GalN-induced acute liver injury, coupled with the activation of the Nrf2 signaling pathway. The evidence encouraged us to explore whether the underlying target of MF is closely associated with KC-derived HO-1 in LPS-D-GalN-mediated fulminant hepatic damage. Overall, the study aimed to search for effective molecule targets of MF against FHF, to elucidate the protective mechanism of MF, and to demonstrate the involvement of HO-1. The study has screened for a potential antidote to LPS/D-GalN-induced acute liver injury and has provided novel insights into the target and mechanisms of MF.

## Materials and Methods

### Animals

BALB/c mice, weighing 18–22 g, were obtained from the Experimental Animal Center of Chongqing Medical University. The animals were kept in individual wire-bottomed cages. All animals were fed a standard laboratory diet and water *ad libitum* and maintained in a controlled environment (20–25°C, 50% ± 5% relative humidity, 12 h dark/light cycle). The animals were acclimatized to the experimental conditions for at least l week before use in experiments. All experimental procedures involving animals were approved by the Animal Care and Use Committee of Chongqing Medical University.

### Reagents

MF (C_19_H_18_O_11_, FW = 422.34, purity ≥ 95%) was purchased from Nanjing ZeLang Medical Technology Co. Ltd. (Nanjing, China). LPS (*Escherichia coli*, 0111: B4), D-GalN, and ZnPP IX were obtained from Sigma (St. Louis, MO, USA). ALT and AST detection kits were purchased from Nanjing Jiancheng Bioengineering Institute (Nanjing, China). The bicinchoninic acid (BCA) protein assay kit was purchased from Pierce (Rockford, IL, USA). Trizol reagent was purchased from Invitrogen (Grand Island, NY, USA). AMV transcriptase, RNasin, SYBR green PCR Master Mix, and the Dual-Luciferase Reporter Assay kit were obtained from Promega (Madison, WI, USA). The tumor necrosis factor (TNF)-α ELISA kit was obtained from Bender MedSystems (Vienna, Austria). Rabbit HO-1 antibody was obtained from Abcam (Cambridge, MA, UK), rabbit β-actin antibody was purchased from Cell Signaling Technology (Boston, MA, USA), and the rat anti-mouse TLR4/MD-2 complex and rat IgG2a isotype control PE were obtained from eBioscience (San Diego, CA, USA). Lipofectamine 2000 was obtained from Invitrogen (Carlsbad, CA, USA).

### Kupffer Cell Isolation and Culture

Liver-resident Kupffer cells were isolated from mice. Briefly, liver samples were perfused with calcium (Ca^2+^)- and magnesium (Mg^2+^)-free Hanks' balanced salt solution (HBSS) containing 0.5 mM EGTA, 10 mM HEPES, and 4.2 mM NaHCO_3_, Type I collagenase (0.05%), and trypsin inhibitor (50 μg/mL) for 10–20 min. The liver samples were removed from the solution and transferred into a sterile tube, minced into small pieces by using scissors, and placed in RPMI 1640 medium supplemented with 5% fetal bovine serum (FBS) and 0.5 mg/mL collagenase at 37°C for 15 min. Then, the liver cell suspensions were filtered through a sterile nylon mesh and placed into fresh, calcium-free medium and subsequently centrifuged at 250 rpm for 15 min. The supernatant was discarded, and the cell pellets were resuspended in RPMI with 5% FBS and separated by centrifugation on a 25–50% Percoll gradient. The Kupffer cells in the interface were seeded in RPMI 1640 containing 10% FBS, 100 μM β-mercaptoethanol, 10 μg/mL insulin, 100 μg/mL streptomycin, and 100 U/mL penicillin at 37°C in an atmosphere of 5% CO_2_.

### Experimental Protocols

FHF was induced in mice by the injection of LPS (10 μg/kg) and D-GalN (700 mg/kg). The mice were gavaged orally with PBS (Control) or MF (30, 100, or 150 mg**/**kg, respectively) twice, at 1 and 7 h prior to LPS/D-GalN injection. ZnPP IX (45 mg/kg) was administered intraperitoneally (i.p.) 30 min before LPS/D-GalN. The survival rate was evaluated for up to 48 h after LPS/D-GalN administration. The serum and liver were collected at the indicated time point for the analysis. For KC depletion, 25 mg/kg GdCl_3_ was intravenously (i.v.) injected into mice at 24 h before challenge or cell transfer. In the adoptive transfer experiment, 2 × 10^6^ donor KCs were i.v.-infused into the tail vein of the recipient mouse in the 24 h before LPS challenge.

In the experiment with KCs, primary KCs were cultured in FBS-free RMPI 1640 and pretreated with PBS or MF (10^−5^ M) for 30 min, and then PBS or LPS (100 ng/mL) was added. The supernatants and cells were collected at 30 min or 3 h after LPS for the subsequent analyses.

### ALT and AST Measurement

Blood was collected from the retro-orbital vein of the eye of mice 6 h after the LPS/D-GalN injection. The blood samples were centrifuged at 3,000 rpm for 10 min. The serum was separated, transferred to other tubes, and frozen at −20°C. ALT and AST activities were assayed by using commercially available kits in accordance with the manufacturer's recommendations.

### Cytokine Analysis

Hepatic tissues and cell supernatants were assayed for TNF-α level by ELISA in accordance with the manufacturer's protocol. Briefly, samples or standards were pipetted into a microplate pre-coated with a monoclonal antibody specific for mouse TNF-α. After any unbound substances had been washed away, an enzyme-linked polyclonal antibody specific for mouse TNF-α was added to each well. After any unbound antibody-enzyme reagent had been washed away, a substrate solution was added to the wells. The enzyme reaction yielded a blue product that turned yellow when the stop solution was added. The intensity of the color was in proportion to the amount of mouse TNF-α bound in the initial step, and the concentration of the sample values were then read from the standard curve.

### Histopathology Analysis

The liver tissue samples from mice were excised and fixed in 10% formaldehyde at 25°C and then embedded in paraffin. Serial paraffin sections (4 μm) were stained with hematoxylin-eosin for conventional morphological evaluation by using a light microscope (Olympus, Tokyo, Japan).

### Reverse Transcription-Quantitative Polymerase Chain Reaction (RT-qPCR)

Total RNA was isolated from the liver and cell samples by using Trizol reagent in accordance with the manufacturer's protocol. First-strand complementary DNA (cDNA) was synthesized. The cDNA samples were then incubated at 90°C for 7 min to stop the reaction. Quantitative PCR was performed with SYBR Green PCR Master Mix in accordance with the protocol. The primers were used to amplify TNF-α cDNA (sense, 5′-TTC TCA TTC CTG CTT GTG GC-3; antisense, 5′-GTT TGC TAC GAC GTG GGC TA-3′), HO-1 cDNA (sense, 5′-GAG ATA GAG CGC AAC AAG CAG-3′; antisense, 5′-CTT GAC CTC AGG TGT CAT CTC-3′), GAPDH cDNA (sense, 5′-CCT GCA CCA CCA ACT GCT TA-3′; antisense, 5′-TCA TGA GCC CTT CCA CAA TG-3′). The generation of specific PCR products was confirmed by melting curve analysis. Relative HO-1 mRNA expression was normalized to GAPDH expression. Data were calculated by using the 2^−ΔΔCt^ method as described by the manufacturer and were expressed as fold increase over the indicated controls.

### Western Blotting Analysis

The protein contents of liver and cell samples were prepared by using a Protein Extraction Kit (20 mM Tris, 150 mM NaCl, 1 mM EDTA, 1 mM EGTA, 1% Triton X-100, 2.5 mM sodium pyrophosphate, 1 mM Na_3_VO_4_, 1 mM β-glycerophosphate, 1 μg/mL leupeptin, and aprotinin). Protein concentrations were determined by using the BCA protein assay kit. Protein extracts (40 μg) were fractionated on 12% polyacrylamide-sodium dodecyl sulfate (SDS) gel and then transferred to nitrocellulose membranes. Non-specific binding to the membrane was blocked by incubation in 5% (w/v) fat-free milk in Tris-buffered saline (TBS) containing 0.05% Tween-20, followed by incubation with a rabbit primary polyclonal antibody at 4°C overnight. The membrane was then treated with horseradish peroxidase-conjugated goat anti-rabbit secondary antibody. Antibody binding was visualized by using a chemiluminescence system and brief exposure of the membrane to X-ray film (Kodak, Japan).

### Flow Cytometry Analysis

KCs were collected, washed with fluorescence-activated cell sorting (FACS) buffer, and blocked with the anti-FcR antibody for 15 min. The cells were then stained by using rat anti-mouse TLR4/MD-2 complex PE or rat IgG2a isotype control PE in the dark at room temperature. Cells were incubated for 30 min, washed twice, and resuspended in 0.5 mL FACS buffer. Fluorescence was measured by flow cytometry and analyzed by using FlowJo software (Tree Star, Inc., Ashland, OR).

### Transfection of Reporter Plasmids and Small Interference RNA (siRNA)

KCs were seeded at 1 × 10^6^ cells per well in 24-well plates and allowed to rest for 1 day before transfection. Cells were transiently transfected with pAP1-Luc or pNF-κB-Luc and the pRL-TK *Renilla* plasmid by using Lipofectamine 2000. The activities of both firefly and *Renilla* luciferases were measured by using the Dual-Luciferase Reporter Assay System according to the manufacturer's instructions. To silence murine HO-1 in primary KCs, pre-confirmed HO-1 specific siRNA and non-targeting control siRNAs were transfected into freshly isolated primary KCs by using Lipofectamine 2000. At 24 h after transfection, the cells were used for subsequent experiments.

### HO-1 Activity Assay

HO enzymatic activity was measured by bilirubin generation, as described previously (26). Briefly, frozen hepatic and cell samples were homogenized in lysis buffer (250 mM Tris·HCl, pH 7.4; 150 mM NaCl; 250 mM sucrose; 0.5 mM PMSF; 1 μg/μL leupeptin; 1 μg/μL aprotinin). The microsomal fraction was obtained by successive centrifugation and washing with 0.15 M KCl, followed by centrifugation (105,000 × *g* for 30 min). The pellet was solubilized in 0.1 M potassium phosphate by sonication and stored at −80°C. The reaction was performed in a mixture containing 2–3 mg/mL protein microsomal fraction, 1 mM glucose-6-phosphate, 0.2 units/mL glucose-6-phosphate dehydrogenase, 0.8 mM NADPH, and 0.025 mg/mL hemin at 37°C for 45 min. After chloroform extraction, the amount of extracted bilirubin was calculated by the difference in absorbance at 464 and 530 nm.

### Statistical Analysis

The study data were expressed as mean ± standard deviation (S.D.). The differences between two groups were calculated by Student's *t*-test, and comparisons of multiple groups were evaluated by one-way analysis of variance (ANOVA), followed by the Tukey *post hoc* test. The survival rates were determined using Kaplan-Meier curves with log-rank tests. *P* < 0.05 were considered to be statistically significant.

## Results

### MF Improved Survival and Pathological Liver Injury Induced by LPS/D-GalN in Mice

**Figure 1:**
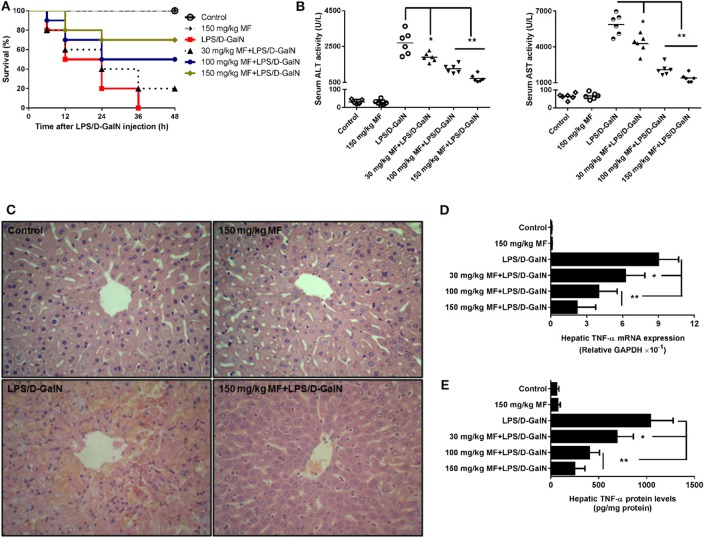
MF improved survival and pathological liver injury induced by LPS/D-GalN. Mice were pretreated orally with vehicle (PBS) or MF (30, 100, or 150 mg/kg, respectively) at 1 and 7 h prior to LPS/D-GalN challenge. Survival rates of mice **(A)** were monitored for 48 h after LPS/D-GalN challenge. Serum ALT and AST activities **(B)** and hepatic tissue pathological changes **(C)** were assessed at 6 h after LPS/D-GalN challenge. Hepatic TNF-α mRNA **(D)** and protein **(E)** were determined by RT-qPCR and ELISA at 1.5 h after LPS/D-GalN challenge. Data are presented as mean ± SD. *n* = 10 (for survival rate analysis) or 6; *P < 0.05 and **P < 0.01.

First, we examined the survival rates induced by LPS/D-GalN in MF-treated and untreated mice. As expected, LPS/D-GalN resulted in high lethality, with 100% mortality occurring within 36 h. Pre-treatment of mice with MF improved survival rates in a dose-dependent manner; 70% of the mice survived to the end of the 48-h observation period in the 150 mg/kg MF-treated group (Figure 1A). With regard to the association of lethality with hepatic damage, we analyzed the liver enzymes and pathological changes in the serum aminotransferases and tissue sections. Serum concentrations of ALT/AST, which are released into the blood upon damage to liver cells by LPS/D-GalN, were markedly lower in MF-treated mice (Figure 1B). Similarly, there were clear improvements in the pathology of the liver after MF treatment (Figure 1C). These results indicated that MF exerts protective activity against LPS/D-GalN-induced mortality and liver injury.

### MF Alleviated Hepatic Expression of TNF-α mRNA and Protein Induced by LPS/D-GalN in Mice

After confirmation of the critical role of TNF-α in endotoxin-induced acute liver injury, we attempted to determine whether MF affected LPS-induced TNF-α production. As shown, the expressions of hepatic TNF-α mRNA and protein was sharply increased in LPS/D-GalN-stimulated mice compared to in control mice; in contrast, the increased expression of TNF-α was dose-dependently decreased by MF treatment (Figures 1D,E, *p* < 0.05), suggesting that MF could inhibit the expression increases of TNF-α mRNA and protein induced by LPS/D-GalN.

### MF Promoted Hepatic HO-1 Expression and Activity in LPS/D-GalN-Challenged Mice

**Figure 2:**
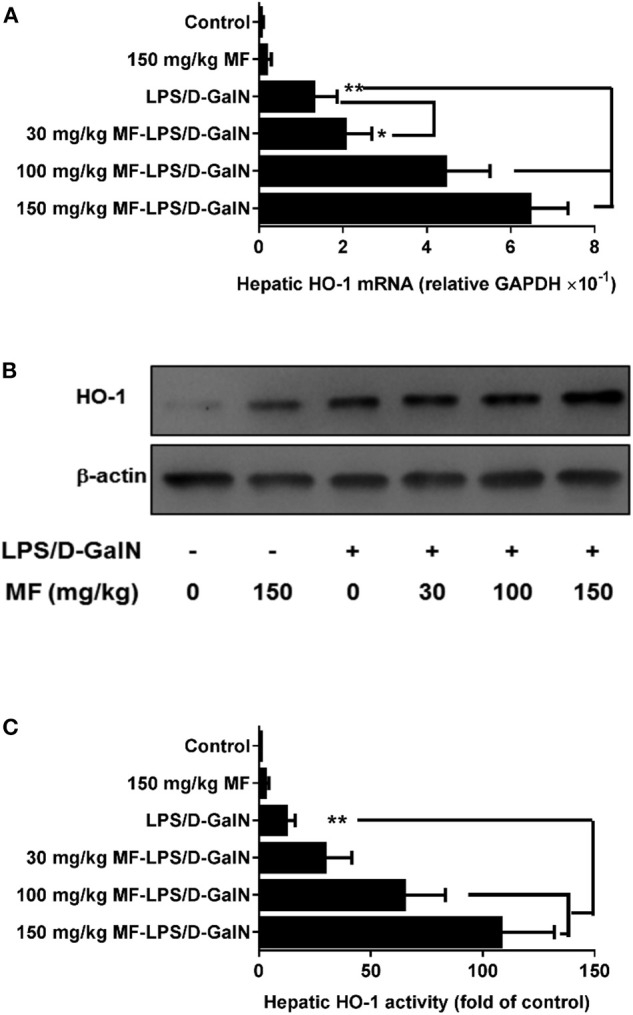
MF upregulated HO-1 expression and activity in LPS/D-GalN-challenged mice. Mice were pretreated orally with vehicle (PBS) or MF (30, 100, or 150 mg/kg, respectively) at 1 and 7 h prior to LPS/D-GalN challenge. Hepatic tissue HO-1 mRNA **(A)** and protein **(B)** expression and activity **(C)** were determined at 6 h after LPS/D-GalN challenge. Data are presented as mean ± SD. *n* = 6, *P < 0.05, and **P < 0.01.

HO-1 is an inducible heat shock protein that participates in cytoprotection. It has been shown that HO-1 exerts a protective effect against LPS/D-GalN-induced FHF. Therefore, we examined whether MF could modify the expression and activity of HO-1. Intriguingly, the expression and activity of HO-1 in hepatic tissues were significantly upregulated by MF treatment compared to those in LPS/D-GalN-treated mice (Figures 2A–C).

### HO-1 Is Responsible for the Effect of MF on LPS/D-GalN-Induced Liver Injury in Mice

**Figure 3:**
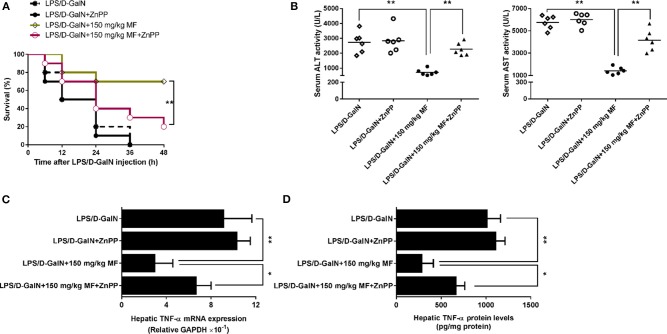
Inhibition of HO-1 removed the protective effect of MF against LPS/D-GalN-induced acute liver injury. Mice were pretreated orally with MF (150 mg/kg) at 1 and 7 h with or without ZnPP IX (40 mg/kg) i.p. 30 min prior to LPS/D-GalN challenge. **(A)** Survival rates of mice were monitored for 48 h after LPS/D-GalN challenge. **(B)** Serum ALT and AST activity were assessed at 6 h after LPS/D-GalN challenge. Hepatic TNF-α mRNA **(C)** and protein **(D)** expression were determined by RT-qPCR and western blotting, respectively, at 1.5 h after LPS/D-GalN. Data were presented as mean ± SD. *n* = 6, *P < 0.05, **P < 0.01.

ZnPP-IX, a widely used HO-1 inhibitor, could suppress HO-1 activity via the competitive conjugation with the porphyrin ring structure owing to its similarity to heme (27, 28). Hence, LPS/D-GalN-treated mice treated with or without MF were exposed to ZnPP-IX. As shown in Figure 3A, ZnPP-IX markedly blunted the improved survival of MF in LPS/D-GalN-treated mice. Likewise, ZnPP-IX blocked the effects of MF on the LPS/D-GalN-induced increases in ALT/AST activities and TNF-α production (Figures 3B–D).

### KCs Are Involved in the Protective Effect of MF on LPS-Mediated Acute Liver Injury in D-GalN-Sensitized Mice

**Figure 4:**
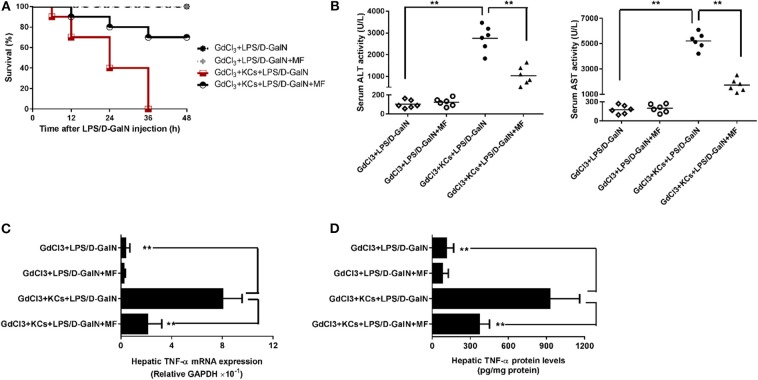
KCs are involved in the protective effect of MF on LPS-mediated acute liver injury in D-GalN-sensitized mice. Mice were injected i.p. with GdCl_3_ 2 days before LPS/D-GalN challenge, KCs were i.v.-infused into mice at 24 h prior to LPS/D-GalN challenge, and MF pretreatment was given orally at 1 and 7 h prior to LPS/D-GalN challenge. **(A)** Survival rates of mice were monitored for 48 h after LPS/D-GalN challenge. **(B)** Serum ALT and AST activities were assessed at 6 h after LPS/D-GalN challenge. Hepatic TNF-α mRNA **(C)** and protein **(D)** expression were determined by RT-qPCR and ELISA, respectively, at 1.5 h after LPS/D-GalN challenge. Data are presented as mean ± SD. *n* = 6, **P < 0.01.

As hepatic TNF-α is produced mainly in inflammatory cells, especially in KCs, we then investigated whether KCs contributed to these effects. We injected mice with GdCl_3_ to selectively deplete KCs in accordance with the previous study (29) and found that this almost completely abrogated LPS/D-GalN-induced mortality and hepatic damage, as indicated by improved survival and decreased serum liver enzymes (Figures 4A,B). Meanwhile, hepatic TNF-α mRNA and protein expression were also sharply diminished (Figures 4C,D). In contrast, adoptive transfer of KCs to GdCl_3_-treated mice restored these effects of the depletion of KCs, suggesting that KCs are required for LPS/D-GalN-induced acute liver injury and TNF-α production. In keeping with those findings, KC depletion could also abolish the protection of MF on LPS/D-GalN-mediated acute liver injury; adoptive transfer of KCs restored the protective effect of MF on LPS/D-GalN-mediated acute liver injury and TNF-α production (Figure 4). Collectively, these data provided compelling evidence that KCs were involved not only in LPS/D-GalN-induced acute liver injury but also in the protective effects of MF on FHF induced by LPS/D-GalN.

### KCs Are Involved in MF-Mediated HO-1 Upregulation in LPS-D-GalN-Treated Mice

**Figure 5:**
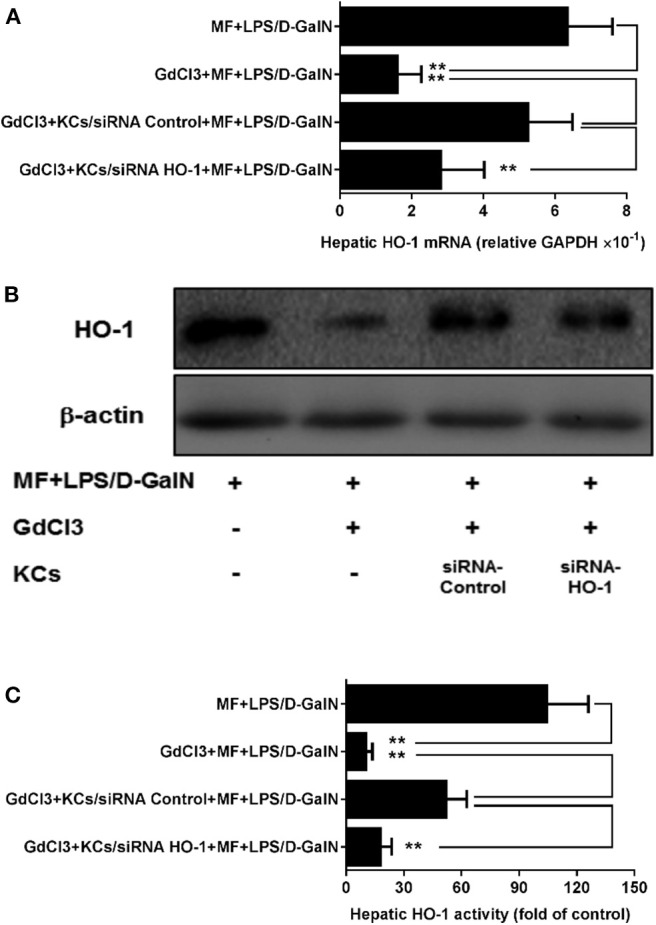
KCs are involved in MF-mediated upregulation of HO-1 in LPS-D-GalN-treated mice. Mice were injected i.p. with GdCl_3_ 2 days before LPS/D-GalN challenge, KCs were i.v.-infused into mice at 24 h prior to LPS/D-GalN challenge, and MF pretreatment was given orally at 1 and 7 h prior to LPS/D-GalN challenge. Hepatic tissues HO-1 mRNA **(A)** and protein **(B)** expression and activity **(C)** were determined at 6 h after LPS/D-GalN challenge. Data are presented as mean ± SD. *n* = 6, **P < 0.01.

To assess whether HO-1 upregulation of MF was involved in KCs, we first used GdCl_3_ to remove KCs in MF-treated LPS-D-GalN-induced mice. As expected, in Figure 5, GdCl_3_ can be seen to have markedly abrogated MF-upregulated HO-1 expression and activity in LPS-D-GalN-treated mice. Further, the adoptive transfer of KCs with control siRNA restored MF-mediated upregulation of HO-1. However, adoptive transfer of KCs with HO-1 siRNA showed an inhibitory effect on HO-1 expression and activity (Figure 5, *p* < 0.01).

### KC-Derived HO-1 Is Key for the Effect of MF in Mice in Response to LPS/D-GalN

**Figure 6:**
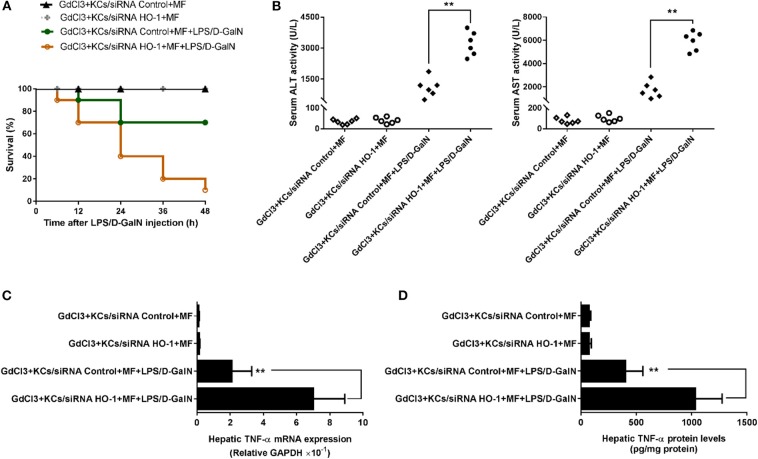
KC-derived HO-1 is critical for the effect of MF against LPS/D-GalN-induced acute liver injury in mice. KCs transfected with HO-1-specific siRNA or non-targeting control were i.v.-infused into mice with KC deletion by GdCl_3_ at 24 h prior to LPS/D-GalN challenge. Mice were pretreated with MF orally at 1 and 7 h prior to LPS/D-GalN challenge. Survival rates **(A)** were evaluated for 48 h following LPS/D-GalN challenge. Serum ALT and AST activities **(B)** were determined at 6 h after LPS/D-GalN challenge. mRNA **(C)** and protein **(D)** expression of TNF-α were measured by RT-qPCR and ELISA. Data are presented as mean ± SD. *n* = 6, **P < 0.01.

Finally, we investigated whether KC-derived HO-1 participated in the protective effects of MF on LPS/D-GalN-mediated acute liver injury in mice. To test this hypothesis, the HO-1 siRNA- or control siRNA-transfected KCs were transferred separately into mice with KC depletion. As shown in Figure 6, transferral of HO-1 siRNA- or control siRNA-transfected KCs into MF alone-pretreated mice with KC depletion did not affect mouse survival rate, ALT/AST activities, or TNF-α production. However, in HO-1 siRNA-knockdown KCs, the protective effects of MF on LPS/D-GalN-induced FHF were reduced compared with those in HO-1 positive KCs, as indicated by the increased death rate, elevated ALT/AST activities, and enhanced TNF-α production (Figures 6A–D, *p* < 0.01). Overall, these results indicated that KC-derived HO-1 was crucial for the effects of MF on LPS/D-GalN-induced FHF.

### MF Inhibited LPS-Activated TLR4 Signaling and TNF-α Production in KCs

**Figure 7:**
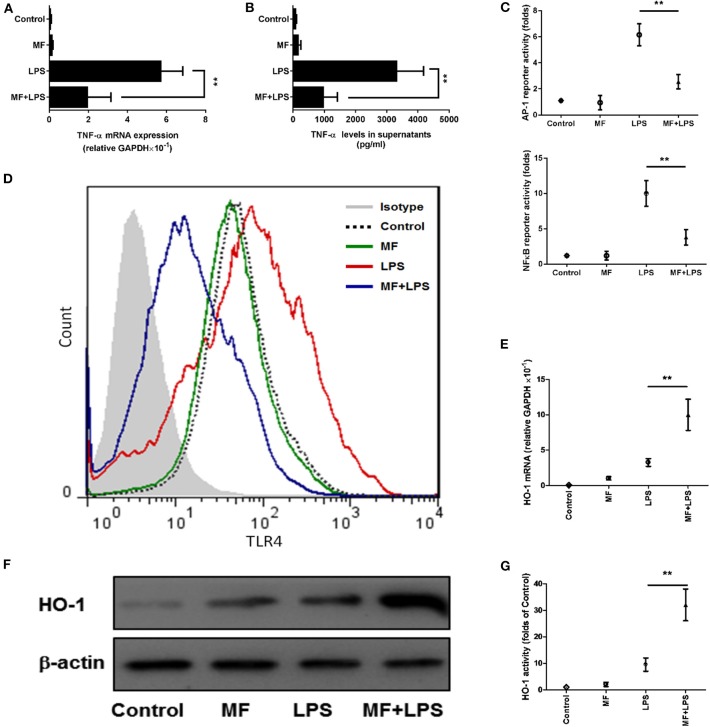
MF inhibited LPS-activated TLR4 signaling and HO-1 upregulation in KCs. KCs were transfected with both pAP1-Luc or pNF-κB-Luc and the pRL-TK *Renilla* plasmid for 24 h, pretreated with PBS or MF (10^−5^ M) 30 min prior to PBS or LPS challenge (100 ng/mL). **(A)** RNA was extracted from KCs at 3 h after LPS challenge and TNF-α mRNA was assayed by real-time RT-PCR. **(B)** Cell culture supernatant was obtained 3 h after LPS stimulation to determine TNF-α levels by using ELISA. **(C)** AP1 and NF-κB activities were measured at 30 min after LPS challenge. **(D)** The expression of TLR4 on the surface of KCs was analyzed at 3 h after LPS by using flow cytometry. HO-1 mRNA **(E)** and protein **(F)** expression and activity **(G)** were determined at 3 h after LPS challenge. Data are presented as mean ± SD. *n* = 6, **P < 0.01.

The observations above suggested that KCs might be a target of the effects of MF in response to LPS. To further verify this hypothesis, we isolated KCs from mice and examined whether MF also has an anti-inflammatory effect on the TNF-α production from KCs stimulated by LPS. Triggering of TLR4 by LPS leads to the activation of transcript factors AP-1 and NF-κB, which promote the expression of TNF-α. The analysis of ELISA and RT-qPCR results revealed that MF remarkably inhibited LPS-induced expression of mRNA and protein TNF-α (Figures 7A,B). Further, the assay of reporter genes showed that MF significantly decreased LPS-induced AP-1 and NF-κB activities (Figure 7C). In parallel with these results, flow cytometry analysis indicated that MF also downregulated LPS-enhanced TLR4 expression on the surface of KCs (Figure 7D). All these data supported the conclusion that MF could effectively suppress TLR4 signaling and TNF-α production triggered by LPS in KCs.

### HO-1 Mediated the Suppression of MF on LPS-Triggered Inflammatory Responses in KCs

**Figure 8:**
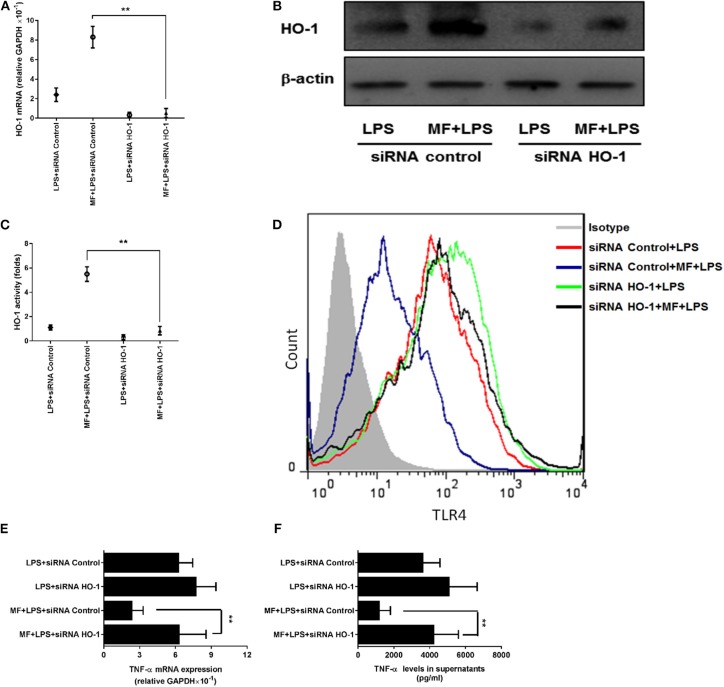
HO-1 mediated the suppression of MF on the LPS-induced inflammatory responses in KCs. HO-1 specific siRNA and non-targeting control siRNA were transfected into freshly isolated primary KCs for 24 h. After transfection, the cells were pretreated PBS or MF (10^−5^ M) 30 min prior to PBS or LPS challenge (100 ng/mL). HO-1 mRNA **(A)** and protein **(B)** expression and activity **(C)** were determined at 3 h after LPS challenge. Total RNA was extracted from KCs at 3 h after LPS challenge and TNF-α mRNA expression was assayed by real-time RT-PCR **(D)**. Cell culture supernatant was obtained at 3 h after LPS challenge and TNF-α protein expression was determined by ELISA **(E)**. The expressions of TLR4 on the surface of KCs was analyzed at 3 h after LPS challenge by flow cytometry **(F)**. Data are presented as mean ± SD. *n* = 6, **P < 0.01.

Given that the inhibitory role of MF in TLR signaling occurs through the HO-1 pathway in KCs, we first assessed the role of MF in the expression and activity of HO-1. Consistent with the above results observed in the animal experiment, the expression and activity of HO-1 were greater in MF-treated KCs than in untreated LPS-stimulated KCs (Figures 7E–G, *p* < 0.01). Further, knockdown of HO-1 with a specific HO-1 small interfering RNA markedly downregulated the HO-1 expression and activity induced by MF in LPS-stimulated KCs (Figures 8A–C). In line with these results, knockdown of HO-1 also abolished the beneficial role of MF on the TLR4 expression and TNF-α production induced by LPS (Figures 8D–F). These data indicated that HO-1 may mediate the anti-inflammatory effect of MF in KCs by downregulating the TLR4 signaling pathway.

## Discussion

Fulminant liver failure is one of the most lethal clinical syndromes and is characterized by rapid deterioration of liver function, which leads to hepatic encephalopathy, systemic inflammatory response syndrome, and even multiorgan failure, which pose severe threats to a patient (4). Liver transplantation remains the only effective therapeutic strategy, but patient recovery is currently constrained by the limitations of organ donation (30). Accordingly, there is an urgent need to find effective drugs to ameliorate FHF and avoid severe secondary complications. In this study, the protective role of MF and the underlying mechanism of action were explored in an LPS/D-GalN-induced mouse model of FHF. Pretreatment with MF significantly enhanced the survival of FHF mice, decreased the activities of ALT and AST in serum, and attenuated liver injury induced by LPS/D-GalN. MF administration not only reduced LPS-mediated TNF-α production but also inhibited KC activation by suppressing the TLR4/NF-κB signaling pathway. Furthermore, the inhibition of TNF-α by MF was indeed associated with the upregulation of HO-1 in KCs.

TNF-α is recognized as a critical inflammatory mediator in the pathogenesis of inflammation and infection-related diseases; moreover, its toxicity may directly damage hepatocytes (31). High amounts of circulating pro-inflammatory TNF-α, the characteristic manifestation of FHF, ultimately induce multiple organ failure (32). TNF-α, via binding TNF receptor on the surface of hepatocytes as an exocellular death signal, can stimulate inner oxidative stress and initiate the apoptotic pathway, which triggers more extensive hepatocyte death and a serious inflammatory response (33). The administration of TNF-α was shown to accelerate liver injury in this model by neutralizing antibodies against TNF-α or inhibitors of TNF-α production; knock-out of TNF-α or TNF-α receptors completely abrogated LPS/D-GalN-induced FHF (34–36). In addition, the production and liberation of TNF-α are predominantly regulated by the TLR4/NF-κB signaling pathway during liver inflammation (37). Accordingly, the inhibition of the TLR4/NF-κB signaling pathway can decrease TNF-α production, which is an effective strategy for FHF alleviation (38, 39). In our study, MF suppressed TNF-α synthesis transcriptionally and translationally, as shown by the decrease in mRNA and protein of TNF-α after MF pretreatment in both mice and KCs. As shown by the reporter gene in KCs, the TLR4 signaling pathway was significantly inhibited by MF, suggesting that MF could reduce TNF-α production via inhibition of TLR4/NF-κB signaling. The abrogation of TNF-α by MF pretreatment significantly retarded the progression of fulminant hepatic damage, which was a critical contribution to the mechanism of MF against acute liver injury.

Although various cell types in the liver are capable of producing TNF-α, KCs are acknowledged as the main source (40). KC-derived TNF-α is majorly involved in the development of FHF (41). Indeed, the liver injury in LPS/D-GalN-induced FHF is associated with activated KCs. The deletion of KCs by GdCl_3_ not only completely blocked this disease but also inhibited TNF-α production (42). In contrast, the infusion of KCs restored the diseased state (43). Given these results, we hypothesized that the beneficial effect of MF on LPS/D-GalN-induced FHF was through the inhibition of KCs to produce TNF-α. To confirm this, we utilized GdCl_3_ to delete primary KCs and subsequently treated LPS/D-GalN-treated mice with MF. KC deletion indeed ameliorated the inflammatory response following LPS/D-GalN administration, although the activities of ALT and AST and the expression of TNF-α were not remarkably different in the mice with or without MF pretreatment. Moreover, after transferring KCs to the KC-deleted mice, the severe hepatic inflammation was restored, and the protective effect of MF was again observed, indicating the key role of KCs in the protection of MF against LPS/D-GalN-induced FHF.

HO-1, a member of the cellular defense system, fulfills a significant function in the elimination of oxidative stress products and the suppression of inflammatory response through multiple pathways. HO-1 exerts a protective effect by catalyzing the oxidative degradation of heme to carbon monoxide (CO), iron, and biliverdin-IXα, which scavenges endogenous injury factors to protect hepatocytes (11). Several studies have reported that HO-1 has a positive protective role in hepatitis and that the downregulation of HO-1 expression aggravated the severity of hepatic injury (44, 45). Consistent with this, it was observed that the expression and activity of HO-1 were elevated by MF in the LPS/D-GalN-treated mice, but the protective role of MF was not observed after the use of an HO-1 inhibitor (ZnPP-IX), which showed that the upregulation of HO-1 was a potential target for preventing the deterioration of hepatic injury. However, HO-1 exhibits distinctive regulatory states in different types of cells in the liver, and not every cell can be a therapeutic target for MF against FHF. Hepatic inflammation develops through cascade amplification (46). Thus, the restrictions of the target cell and the superior inflammatory response can fundamentally block the development of liver injury from the source. Activation of KCs has a clear link with the development of hepatic inflammation (47). To confirm this, KCs were examined, and the role of HO-1 in KCs was also investigated. MF pretreatment promoted the expression and activity of HO-1 in KCs, but the protection of MF was equally abated when KC-derived HO-1 expression was silenced by siRNA. Furthermore, when KCs with HO-1 or control siRNA were transferred to primary KC-deleted mice that were subjected to LPS/D-GalN challenge, the survival rate and the activities of transaminases were not improved, and meanwhile the high level of TNF-α. These data more comprehensively illuminated that the upregulation of KC-derived HO-1 was imperative for the attenuation of acute liver injury by MF.

In conclusion, these data revealed that MF exhibited significant protection against LPS/D-GalN-induced acute liver injury and alleviated TNF-α production through the inhibition of the TLR4/NF-κB signaling pathway. The abrogation of TNF-α was correlated with the upregulation of HO-1 expression. Furthermore, KCs played a key role in the protection of MF against acute liver injury and the promotion of KC-derived HO-1 was involved in the protective effects of MF. Collectively, MF attenuated acute liver injury induced by LPS/D-GalN through a reduction in TNF-α production by promoting KCs to upregulate HO-1 expression. This study has demonstrated a potential prophylactic agent with a novel mechanism of action against LPS/D-GalN-induced acute liver injury.

## Data Availability Statement

The raw data supporting the conclusions of this article will be made available by the authors, without undue reservation, to any qualified researcher.

## Ethics Statement

The animal study was reviewed and approved by the Animal Care and Use Committee of Chongqing Medical University. Written informed consent was obtained from the owners for the participation of their animals in this study.

## Author Contributions

SY and GK performed experiments, analyzed data, and wrote the manuscript. SW, ZZ, and XY performed experiments and analyzed data. LZ and BW provided guidance and suggestions for the study. XG and JW conceived and supervised the study, designed experiments, analyzed and interpreted data, and wrote the manuscript.

### Conflict of Interest

The authors declare that the research was conducted in the absence of any commercial or financial relationships that could be construed as a potential conflict of interest.
